# Estimating survival and choosing treatment for spinal metastases: Do spine surgeons agree with each other?

**DOI:** 10.1016/j.jor.2021.11.015

**Published:** 2021-11-27

**Authors:** Quirina C.B.S. Thio, Nuno Rui Paulino Pereira, Olivier van Wulfften Palthe, Daniel M. Sciubba, Jos A.M. Bramer, Joseph H. Schwab

**Affiliations:** a Department of Orthopaedic Surgery, Amsterdam University Medical Center Amsterdam, Meibergdreef 9, 1105AZ, Amsterdam, the Netherlands; b Division of Orthopaedic Oncology, Department of Orthopaedics Massachusetts General Hospital – Harvard Medical School, 55 Fruit Street, Boston, MA, 02114, USA; c Department of Neurosurgery, The Johns Hopkins Hospital – the John Hopkins University School of Medicine, 1800 Orleans St, Baltimore, MD, 21287, USA

**Keywords:** Bone metastasis, Spine, Survival estimation, Survey

## Abstract

**Purpose:**

This study aimed to investigate spine surgeons’ ability to estimate survival in patients with spinal metastases and whether survival estimates influence treatment recommendations.

**Methods:**

60 Spine surgeons were asked a survival estimate and treatment recommendation in 12 cases. Intraclass correlation coefficients and descriptive statistics were used to evaluate variability, accuracy and association of survival estimates with treatment recommendation.

**Results:**

There was substantial variability in survival estimates amongst the spine surgeons. Survival was generally overestimated, and longer estimated survival seemed to lead to more invasive procedures.

**Conclusions:**

Prognostic models to estimate survival may aid surgeons treating patients with spinal metastases.

## 1 Introduction

Spinal metastases frequently occur in patients with cancer.^1^ Around 60% of patients with terminal cancer have bone metastases and these are most often found in the spine. Spinal metastasis may lead to pain, fractures, and neurological symptoms, sometimes even, paralysis due to spinal cord compression.^2^ The goal of palliative treatment in patients with bone metastases is to reduce pain and maintain or improve function. There are several treatment options.^3^^,^^4^ Therapeutic agents such as denosumab have been shown to reduce the risk of skeletal related events in patients with bone metastasis.^5^^,^^6^ Radiotherapy is usually used in patients with a spinal metastasis, mostly for pain control and improvement or prevention of neurological compromise.^7^^,^^8^ Stereotactic radiosurgery delivers precisely-targeted radiation in higher and fewer dosages than conventional radiotherapy. Surgical options range from minimal invasive procedures such as vertebroplasty to en-bloc resection of an affected spinal segment, with the last procedure leading to a longer rehabilitation period and higher risk of complications. In general the goals of surgery are: stabilization of the spine, decompression in case of spinal cord compression, and local tumor control. In addition, a histological diagnosis can be obtained if necessary. A physician should always balance the benefits of surgery against the risk for complications, and have an expectation that the patient may outlive the rehabilitation period in order to benefit from surgery.^1^^,^^9^^,^^10^ Variation in patient and tumor characteristics and symptomatology mandates individualized treatment. Life expectancy is considered an important factor when discussing treatment options for a patient with spinal metastases. Multiple prognostic models have been developed that estimate survival in patients with spinal metastases.^11^, ^12^, ^13^, ^14^, ^15^, ^16^ However, it is unclear whether these models are needed. In this study we investigate how well surgeons estimate survival in patients with spinal metastasis and whether treatment recommendations are influenced by estimated survival.

Our study questions are: 1) What is the surgeons' survival estimate variability? (2) What is the accuracy of surgeons’ survival estimates? (3) Do survival estimates influence the choice of treatment for patients with spine metastases?

## 2 Methods

### 2.1 Study design and setting

**Fig. 1:**
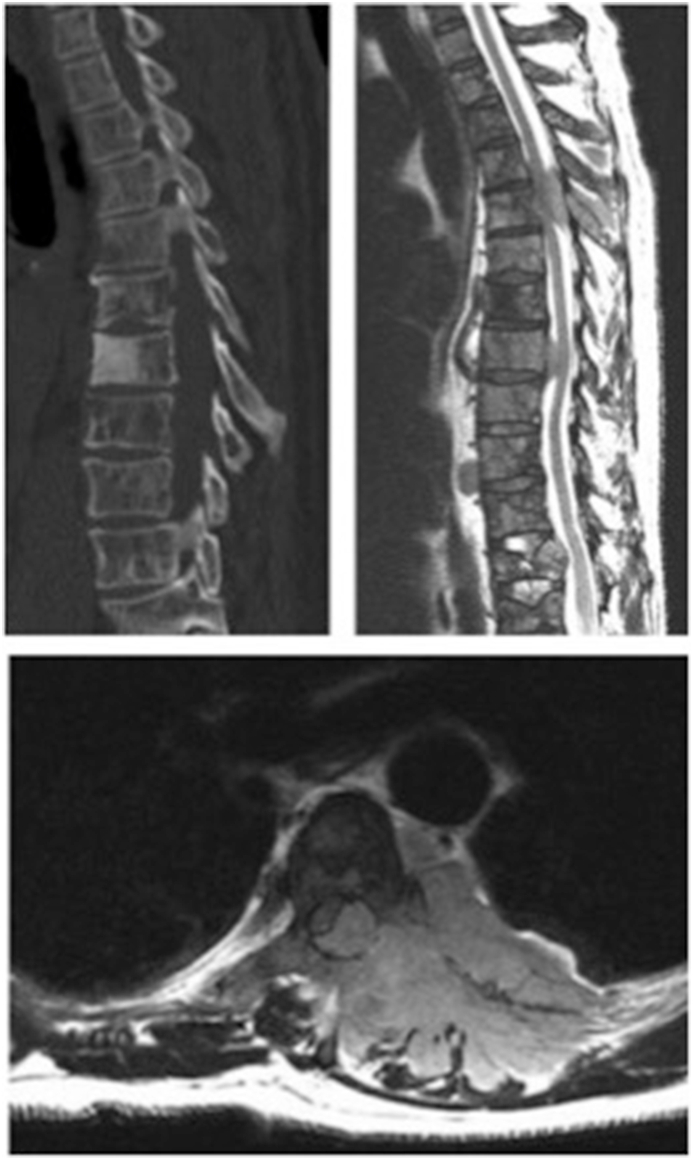
Example of the presentation of a case (case 1). 1) What is the estimated life expectancy for this patient (in months)?. 2) What treatment (en-bloc spondylectomy, decompression and/or stabilization, kyphoplasty/vertebroplasty, palliative radiotherapy, stereotactic radiosurgery, no local therapy) would you recommend for the spinal lesion?. This 57-year-old man was diagnosed with multiple myeloma 5 years ago. He presents with a chief complaint of bladder incontinence and saddle anesthesia over the past week, and lower extremity weakness over the past two days. His back pain is relieved when lying down, and he uses a cane/walker to ambulate because of lower extremity weakness (ASIA D). The patient has no brain metastases, and no visceral metastases. He received radiotherapy to the affected region of the spine (30 Gy), and was treated with chemotherapy. He presents with the above images –mass extending from T4 to T6. The workup laboratory values are: white blood cell count 7.1 × 103/mm3, hemoglobin level 8.7 g/dL, platelet count 368 × 103/mm3, creatinine level 1.07 mg/dL, and calcium level 8.1 mg/dL.

This cross-sectional case vignette survey study was approved by our institutional review board. In total 12 cases of different patients with a spinal metastasis were collected and presented to participating spine surgeons. Surgeons were asked two questions per case: 1) “What is the estimated life expectancy for this patient (in months)?”, and 2) “What treatment would you recommend for the spinal lesion? (en-bloc spondylectomy, decompression and/or stabilization, kyphoplasty/vertebroplasty, palliative radiotherapy, stereotactic radiosurgery, no local therapy)”. The following variables were described for each case: age, sex, cancer type, time since primary tumor diagnosis, chief complaint, ambulatory status, American Spinal Cord Injury Association (ASIA) score (a scale used or neurologic assessment), presence of additional bone metastases, presence of visceral metastases (lung or liver), presence of brain metastases, previous treatment with local radiotherapy, previous chemotherapy, white blood cell count, hemoglobin level, platelet count, creatinine level, and calcium level (Table 1). Most of these factors were found to be associated with survival in previous studies.^11^, ^12^, ^13^, ^14^, ^15^, ^16^ Each case was presented with a sagittal image of the computed tomography (CT) scan and a sagittal and axial image of the magnetic resonance imaging (MRI) scan of the spinal lesion (Fig. 1). Surgeons were asked not to use existing prediction models.

**Table 1:** Overview of the cases that were used for the survey.

Case	Sex	Age	Cancer	Tumor region	ASIA classification*	Other metastases	Actual survival category	Exact survival
1	Male	57	Multiple myeloma	Thoracic	D	None	≤3 Months	1,2 Months
2	Male	69	Prostate	Thoracic	D	Bone	≤3 Months	3,0 Months
3	Male	64	Multiple myeloma	Thoracic	D	None	3–12 Months	5,6 Months
4	Male	62	Multiple myeloma	Cervical	A	Brain	≥12 Months	20,4 Months
5	Male	52	Lung	Thoracic	D	Bone	3–12 Months	5,5 Months
6	Female	74	Lung	Thoracic	C	Bone	≤3 Months	1,0 Months
7	Male	42	Renal cell	Lumbar	E	None	3–12 Months	6,2 Months
8	Male	60	Lung	Cervical	D	None	≥12 Months	12,5 Months
9	Female	63	Breast	Cervical	D	Bone	≥12 Months	72,8 Months
10	Female	66	Renal cell	Thoracic	D	None	≥12 Months	13,0 Months
11	Male	59	Renal cell	Thoracic	C	Bone, visceral and brain	≤3 Months	1,7 Months
12	Male	79	Prostate	Thoracic	C	Bone and visceral	3–12 Months	4,9 Months

*ASIA classification: ASIA A = complete impairment, ASIA B = No motor function. Sensory is preserved below the neurologic level and extends through sacral segments S4–S5, ASIA C = Preserved motor function below the neurologic level, with a muscle grade less then three for most key muscles below the neurologic level, ASIA D = Preserved motor function below the neurologic level, with a muscle grade of three or more for most key muscles below the neurologic level have, ASIA E = no impairment.

The cases including the questions were uploaded on the web-based assessment tool SurveyMonkey (Palo Alto, CA, USA).

### 2.2 Participants

Participants were recruited between May 2016 and August 2017. We invited all members (n = 127) of the Skeletal Oncology Research Group (SORG), an international collaborative in musculoskeletal oncology which consists of specialist from different medical fields who are involved in musculoskeletal oncology.^17^ We furthermore reached out to all members of the Dutch Spine Society (n = 163), a collaborative consisting of Dutch orthopedic surgeons and neurosurgeons involved in surgery of the spine. We also encouraged participants to invite their colleagues and acquaintances involved in the management of spinal metastases. Acknowledgement and scientific curiosity were the only incentives for study participation.

### 2.3 Variables and outcome measures

All participants were asked about their specialty [orthopaedic surgery or neurosurgery], what year they finished residency, how many metastatic spine cases they treat annually [<10 cases, 11–20 cases, 21–40 cases, >40 cases], and country of practice.

Our primary outcome was variability of survival estimates between surgeons. We also assessed accuracy of the surgeons’ survival estimates. Finally, we assessed the association between survival estimate and treatment recommendation [en-bloc spondylectomy, decompression and/or stabilization, kyphoplasty/vertebroplasty, palliative radiotherapy, stereotactic radiosurgery, no local therapy].

### 2.4 Statistical analysis, study size

Categorical variables were described as frequencies with percentages. There were no missing values.

The Intraclass Correlation Coefficient (ICC) with 95% Confidence Interval was calculated through a two-way mixed-effects model. The ICC is calculated by comparing the variability of ratings per subject with the total possible variability in ratings, and ranges from 0 to 1; with 1 indicating perfect agreement and 0 indicating no agreement.^18^ A score of less than 0.5 reflects poor agreement, between 0.5 and 0.75 moderate, between 0.75 and 0.90 good and above 0.90 excellent.^19^Stata® 13.0 (StataCorp LP, College Station, TX, USA) was used for all statistical analysis.  

An ante-hoc power calculation determined that at least 48 participants were needed to find an ICC of 0.2 (or greater) for variation in survival estimation in 12 cases (alpha 0.05, power 0.80).

## 3 Results

### 3.1 Demographics of participants

Out of 60 participants that completed the online survey, 49 were orthopedic surgeon (82%) and 11 were neurosurgeon (18%) (Table 2). Of these participants, 28% treated more than 40 cases yearly, 25% treated 21–40 cases, 22% treated 11–20 cases, and.25% treated less than 10 cases. Most observers practiced in North America (60%), followed by Europe (32%).

**Table 2:** Observer demographics from spine surgeons (n = 60).

	n (%)
Subspecialty	
Orthopedic surgeon	49 (82%)
Neurosurgeon	11 (18%)
Years after finishing residency	
<5 years	24 (40%)
5–10 years	15 (25%)
>10 years	21 (35%)
Cases treated per year	
<10 cases	15 (25%)
11-20 cases	13 (22%)
21-40 cases	15 (25%)
>40 cases	17 (28%)
Continent of practice	
North America^a^	36 (60%)
Europe^b^	19 (32%)
Other^c^	5 (8%)

a Consisting of United States of America (n = 35) and Canada (n = 1).

b Consisting of The Netherlands (n = 17), Belgium (n = 1), and Italy (n = 1).

c Consisting of China (n = 2), Japan (n = 2), and Argentina (n = 1).

### 3.2 Estimated survival variability

The intraclass correlation for estimating life expectancy was 0.22 (95% CI 0.12–0.48), reflecting poor interobserver agreement (Table 3). When we stratified by actual survival category (≤3 months, 3–12 months, ≥ 12 months), surgical specialty, years after finishing residency, numbers of cases treated annually, and continent of practice, the ICC was similar. The estimations for case 1 ranged from 3 to 90 months, for case 2 from 6 to 120 months, for case 3 from 6 to 90 months, for case 4 from 1 to 120 months, for case 5 from 2 to 60 months, for case 6 from 1 to 36 months, for case 7 from 6 to 180 months, for case 8 from 3 to 60 months, for case 9 from 3 to 180 months, for case 10 from 3 to 100 months, for case 11 from 2 to 50 months and for case 12 from 3 to 120 months.

**Table 3:** Overall Intraclass Correlation Coefficient (ICC) and ICC stratified by survival category.

	Individual	
Subjects	ICC	95% CI
Overall	0.22	0.12–0.46
*By survival category*		
≤3 Months	0.26	0.10–0.84
3–12 Months	0.25	0.09–0.82
≥12 Months	0.22	0.08–0.15
*By surgical specialty*		
Orthopaedic surgery	0.21	0.11–0.45
Neurosurgery	0.24	0.10–0.53
By number of cases		
<10 cases	0.27	0.13–0.54
11-20 cases	0.29	0.14–0.56
21-40 cases	0.21	0.09–0.47
>40 cases	0.22	0.10–0.47
*By years since residency*		
<5 years	0.20	0.09–0.43
5–10 years	0.23	0.10–0.49
>10 years	0.26	0.13–0.52
*By continent*		
North America	0.20	0.10–0.43
Europe	0.24	0.12–0.51
Other	0.31	0.10–0.63

### 3.3 Accuracy of survival estimates

**Fig. 2:**
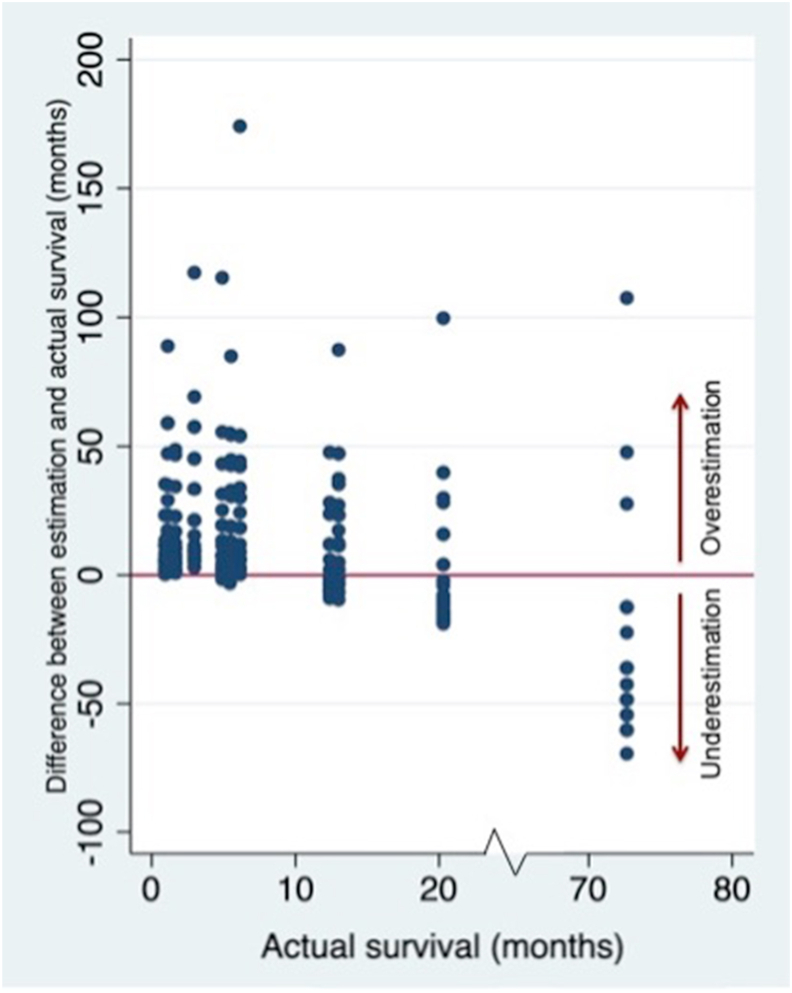
The difference between estimated survival and actual survival plotted on the y-axis, with actual survival on the x-axis. The red line represents the situation in which the estimated survival is the same as the actual survival. (For interpretation of the references to colour in this figure legend, the reader is referred to the Web version of this article.)

The estimated median of cases with an actual survival of ≤3 months was 12 (IQR 6–24). Of the cases with an actual survival of 3–12 months the median was also 12 (IQR 7–36) and of the cases with an actual survival of ≥12 months it was 15 (IQR 9–36). Fig. 2 shows the absolute difference between the actual survival categories and the estimations. As shown in Table 4, the estimations were mostly higher than the actual survival. For cases with an actual survival of ≤3 months, the median difference was 9 months (IQR 4–21), for cases with an actual survival of 3–12 months the median difference was 7 months (IQR 2–30) and for cases with an actual survival of ≥12 months the median difference was −6 (IQR -17-11).

**Table 4:** The survival estimations per case, from lowest actual survival to highest.

Case	Exact survival (months)	Median difference (months)	Min. – max. difference (months)
6	1	5	0–35
1	1	16	12–89
11	2	4	0–48
2	3	21	3–17
12	5	7	−2–115
5	6	0	−3–55
3	6	18	0–84
7	6	18	0–174
8	13	0	−9–48
10	13	11	−10–87
4	20	−14	−19–99
9	73	−49	−70–107

### 3.4 Estimated survival and choice of treatment

**Fig. 3:**
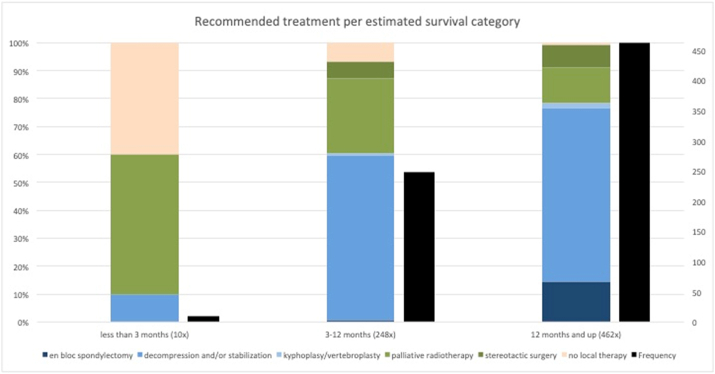
A visualization of the recommended treatment by estimated treatment. For this figure, the survival estimates were grouped in three categories (less than 3 months, 3–12 months, and more than 12 months). The black bars show the absolute numbers.

Overall, decompression and/or stabilization was chosen 434 times (60%), palliative radiotherapy 130 times (18%), en bloc spondylectomy 69 times (10%), stereotactic surgery 52 times (7%), no local therapy 10 times (3%), and kyphoplasty/vertebroplasty 10 times (1%). Fig. 3 shows the distribution of recommended treatment per estimated survival category. Local therapy and palliative radiotherapy were mostly chosen if estimated survival was low, whilst a form of surgery was most frequently chosen if estimated survival was high.

Surgeons that practiced in the United States recommended decompression and/or stabilization more often than surgeons that practiced in Europe, while surgeons that practiced in Europe more often chose en bloc spondylectomy and palliative radiotherapy (p = 0.02).

## 4 Discussion

Survival estimation is crucial for patients with spinal metastases. Choosing the appropriate treatment option, ranging from the decision not to treat to invasive surgery, relies on many factors. One of the most important factors is the patient's expected survival.^1^^,^^9^^,^^10^^,^^20^ Furthermore, physicians can use to inform their patients, either by directly giving them insight in their prognosis, which some may want, or by focusing on the morbidity associated with surgery when a patient's life expectancy is low.^21^^,^^22^

Several prognostication models have been developed and validated to help the surgeon estimating survivals.^12^, ^13^, ^14^, ^15^, ^16^^,^^23^ This study aimed to provide some insight in surgeon's ability to estimate survival without using these prognostication models and to assess whether survival estimation was associated with choice of treatment.

### 4.1 Estimated survival variability and accuracy of survival estimates

We found poor agreement between surgeons when estimating survival for patients with spinal metastasis. This was found across the board and was unrelated to years of practice or number of cases treated per year. Survival estimations were overly optimistic in the majority of the cases. There were two cases with a survival of more than 20 months, and on those the was survival underestimated. Our results confirm the findings of Cheon et al. who assessed the clinicians' accuracy to predict survival in patients with advanced cancer in their study.^24^ They included fifteen studies in their review, and found that clinicians' survival predictions were often inaccurate, and clinicians overestimated their patient's survival in the majority of the cases. In our study the observers were even far more inaccurate in their estimations than the studies described in the review. One of the main explanations is the great variability there is in patients with spinal metastasis. The studies in the review each focused on one type of primary tumor, while patients with a spinal metastasis can have different primary tumors. This leads to a big variation in median survival, which makes survival prediction extremely challenging.

### 4.2 Estimated survival and choice of treatment

Existing guidelines recommend to refrain from treatment when life expectancy is lower than two or three months and to perform more invasive procedures when life expectancy is higher.^7^^,^^8^ In our study, surgeons showed a tendency to recommend more aggressive procedures when survival estimates were higher, choosing local therapy and palliative radiotherapy when the survival was estimated under 3 months an more surgical procedures when the survival as estimated more than 12 months. But the estimations were not equally distributed (as seen in Fig. 3), so we can not draw any hard conclusion from our data.

### 4.3 Limitations

We acknowledge that a number of limitations have to be taken into account when interpreting our results. First, these cases are presented on paper. In clinical practice when a surgeon actually sees the patient, he/she might be unknowingly influenced by other factors, such as appearance, that we could not include in our survey. This could be overcome by exposing all surgeons to the patient in real life (which would be logistically challenging and unethical). Second, the survey consisted of 12 cases only with great variance in presentation. It may have been better to include many more cases to assess the accuracy of the surgeons. However, doing this would take a lot more of the observers' time, and it would not be realistic to do so. Third, most observers were from the USA and Europe. Hence, our results may not apply to observers in other parts of the world. Patients and treatment options in other parts of the world might differ, and cultures may have different views on how to treat such patients. It would be interesting to have information on this in the future. Despite these limitations, we believe our results clearly show the shortcomings of surgeons’ survival estimations and the need for prognostic algorithms in patients with spinal metastasis.

## 5 Conclusions

There is poor agreement when it comes to survival estimation between surgeons that treat patients with spinal metastasis. While survival estimation seems to be associated with treatment choice, surgeons overestimate the patients’ survival considerably. Validated algorithms that estimate survival in this group of patients may aid in overcoming this matter and help in surgical decision-making.

## Conflicts of interest

None.

## Ethical review committee statement

Our institutional review board approved of this study.

## Location

This research was performed at the Massachusetts General Hospital in Boston, MA, United States and at the Amsterdam University Medical Center, The Netherlands.

## Disclosures

The authors have nothing to disclose.

## Funding

No external funding was sought for this study.

## Authors’ contribution

Quirina C·B.S. Thio: Study conception and design - data collection – data analysis – drafting article – revision of article. Nuno Rui Paulino Pereira: Study conception and design - data analysis – revision of article. Olivier van Wulfften Palthe: Data acquisition – revision of article. Daniel Sciubba: Study design – data collection – revision of article. Jos A.M. Bramer: Interpretation of the data - revision of article. Joseph H. Schwab: Study conception and design – interpretation of the data – revision of the article.

All authors have reviewed and agreed to their individual contributions prior to submission.

All authors have reviewed and agreed to their individual contributions prior to submission. All authors report no conflicts of interest related to this work.
